# 长春瑞滨联合异环磷酰胺三线以后治疗晚期非小细胞肺癌的疗效和不良反应

**DOI:** 10.3779/j.issn.1009-3419.2015.06.04

**Published:** 2015-06-20

**Authors:** 阳 周, 燕 徐, 静 赵, 巍 钟, 孟昭 王

**Affiliations:** 100730 北京，中国医学科学院中国协和医科大学北京协和医院呼吸内科 Department of Respiratory, Peking Union Medical College Hospital, Chinese Academy of Medical Sciences, Beijing 100730, China

**Keywords:** 肺肿瘤, 长春瑞滨, 异环磷酰胺, Vinorelbine, Ifosfamide, Lung neoplasms

## Abstract

**背景与目的:**

晚期非小细胞肺癌（non-small cell lung cancer, NSCLC）患者根据指南应接受一线和二线的标准治疗，但出现疾病进展后的三线及三线以上治疗没有推荐，医师根据自己的经验给予治疗。本研究观察了长春瑞滨联合异环磷酰胺在晚期NSCLC三线及三线以上治疗的疗效和不良反应。

**方法:**

回顾近4年北京协和医院住院使用长春瑞滨联合异环磷酰胺治疗的患者，满足以下条件：经细胞学或组织病理学证实的NSCLC，既往至少2个化疗或分子靶向治疗后进展，临床上有可测量病灶，美国东部肿瘤协作组（Eastern Cooperative Oncology Group, ECOG）体能状态评分（performance status, PS）0分-2分，无血液系统、肝肾功能异常。按照世界卫生组织（World Health Organization, WHO）标准评价患者近期疗效，根据不良事件分级标准（Common Terminology Criteria for Adverse Events, CTCAE）V4.03标准评价不良事件，随访无疾病进展时间及生存期。

**结果:**

符合条件患者41例，共进行150周期化疗，其中23周期（15.3%）出现用药推迟或剂量调整。部分缓解3例（7.3%），病情稳定25例（61.0%）。中位无进展生存时间5.5个月，中位生存期为10.5个月。血液学异常是最常见的不良反应，3度/4度中性粒细胞下降10.7%，白细胞下降8.7%，血红蛋白下降8.7%。3度/4度非血液学不良反应少见。所有不良反应可以控制，无相关死亡事件。

**结论:**

长春瑞滨联合和异环磷酰胺方案在晚期NSCLC三线及三线以上治疗中安全性较好，同时多数患者可获得一定疗效，但需要进一步大样本的临床试验来证实可否延长总生存期。

## Abstract (en)

**Background and objective:**

Patients with locally advanced or metastatic non-small cell lung cancer (NSCLC) should be treated with first-line and second-line chemotherapy or target therapy according to the lung cancer guidelines. However, no recommended treatments are available after failure to these treatments. In our study, the efficacy and safety of combined chemotherapy of vinorelbine and ifosfamide as third-line therapy or beyond for advanced NSCLC was evaluated.

**Methods:**

A total of 41 patients took a combination of vinorelbine and ifosfamide as third-line or above chemotherapy in Peking Union Hospital between May 2010 and May 2014. They were pathologically or cytologically confirmed as having locally advanced or metastatic NSCLC. Clinically measurable lesions existed, and Eastern Cooperative Oncology Group performance status ranged from 0 to 2. No serious complications were observed in the hematopoietic system, and liver and renal functions were normal.

**Results:**

The 41 patients enrolled in this study were given 150 cycles of chemotherapy, of which 23 cycles (15.3%) were delayed or dose-adjusted. Results of the objective response evaluation showed that 3 cases had objective partial response (7.3%) and 25 cases were stable (61.0%). The median progression-free survival time was 5.5 months, and the median overall survival time was 10.5 months. Hematological toxicity was the most frequent adverse event. Grade 3/4 neutropenia had 10.7% occurrence; grade 3/4 white blood cell decrease had 8.7%; and grade 3/4 anemia had 8.7%. All adverse events were manageable, and no drug-related death was found.

**Conclusion:**

Vinorelbine and ifosfamide as third-line treatment and beyond of NSCLC were well tolerated. Most patients can benefit from this combined chemotherapy. A clinical trial with large samples is necessary to prove whether overall survival will.

肺癌是癌症导致死亡的首要病因，其中非小细胞肺癌（non-small cell lung cancer, NSCLC）约占所有肺癌的80%，且诊断时大多数已为晚期。化疗是晚期NSCLC治疗的基石，可以明显延长患者的生存时间，缓解肺癌相关的症状。根据中国肺癌治疗指南，推荐患者接受一线含铂的两药联合方案和二线培美曲塞或多西紫杉醇单药化疗，对于有表皮生长因子受体（epidermal growth factor receptor, EGFR）基因突变或*ALK*基因重排的患者尽早接受EGFR酪氨酸激酶抑制剂（tyrosine kinase inhibitor, TKI）或间变性淋巴瘤激酶（anaplastic lymphoma kinase, ALK）抑制剂治疗^[1, 2]^。但是部分患者接受上述标准治疗后出现疾病进展，而且体能评分较好，这种情况下指南没有作出推荐，医师根据自己的经验给予治疗。本研究对这部分患者进行了临床探索，回顾性分析长春瑞滨（Vinorelbine, NVB）联合异环磷酰胺（Ifosfamide, IFO）方案三线和三线以上治疗晚期NSCLC的疗效和安全性。

## 1 资料和方法

### 1.1 病例选择

回顾性分析2010年5月-2014年5月北京协和医院呼吸内科收治的晚期NSCLC患者，均接受长春瑞滨联合异环磷酰胺方案的治疗。所有患者均符合如下条件：年龄18周岁以上；经细胞学或组织病理学确诊的NSCLC；根据国际肺癌研究协会（International Association for the Study of Lung Cancer, IASLC）2009年修订的国际肺癌分期方法判定为Ⅲb期或Ⅳ期；既往接受过2个或2个以上的化疗方案，其中包括一个为两药联合含铂方案，可接受过EGFR-TKI治疗（同步放化疗计为一个化疗方案）；美国东部肿瘤协作组（Eastern Cooperative Oncology Group, ECOG）体能状态（performance status, PS）评分为0分-2分；临床上存在可测量病灶[根据世界卫生组织（World Health Organization, WHO）标准]；无合并严重的感染或血液系统、肝肾功能、心肺功能、神经系统异常。

### 1.2 剂量和用法

长春瑞滨联合异环磷酰胺方案（NI方案）的具体用法：长春瑞滨（诺维本，Novelbine，法国皮尔法伯公司）25 mg/m^2^静脉输液快速滴注，第1天和第8天；异环磷酰胺1.2 mg/m^2^静脉输液，第1至3天给药，同时第0、4、8 h予美司钠0.4 g静脉入壶解救，以预防出血性膀胱炎。给药同时予止吐抗过敏治疗。21天为一个治疗周期，4个-6个周期。

### 1.3 疗效和不良反应

每完成2周期化疗后评价近期疗效。根据WHO实体瘤疗效评价标准（Response Evaluation Criteria in Solid Tumor 1.1, RECIST 1.1），分为完全缓解（complete response, CR）、部分缓解（partial response, PR）、病情稳定（stable disease, SD）和疾病进展（progressive disease, PD）。疾病控制率（disease control rate, DCR）为（CR+PR+SD）/全部病例数×100%。总缓解率（overall response rate, ORR）又称有效率为（CR+PR）/全部病例数×100%。记录每周期化疗中不良事件，包括药物使用过程中或使用后任何不适症状、体征或化验异常。其中与化疗药物存在因果相关的记为不良反应。无进展生存期（progression free survival, PFS）定义为从使用NI方案化疗第1周期第1天至疾病进展或死亡时间的随访时间（月）。总生存期（overall suivival, OS）定义为患者从使用NI方案化疗第1周期第1天至死亡或尚未死亡末次随诊时间（月）。

### 1.4 随诊

通过门诊随访或电话随访。随访时间至患者死亡或2014年11月30日为止。

### 1.5 统计学方法

采用SPSS 17.0软件，采用*Kaplan-Meier*方法计算中位PFS和中位OS，并绘制生存曲线。以*P* < 0.05为差异有统计学意义。

## 2 结果

### 2.1 患者一般临床特征

符合条件患者总例数41例，患者的一般临床特征见表 1。中位年龄58岁（范围32岁-75岁）。转移部位包括：对侧肺叶12例，胸腔积液15例，远处淋巴结转移14例，骨转移10例，脑转移6例。

**1:** 患者一般临床特征 Clinical characterisics of 41 patients

Factor	*n*	Proportion (%)
Gender		
Male	27	65.8
Female	14	34.2
Age (yr)		
≥60	18	43.9
< 60	23	56.1
ECOG PS		
0	29	70.7
1	9	22.0
2	3	7.3
Pathological type		
Squamous	15	36.6
Adenocarcinoma	22	53.7
Unknown	4	9.8
Differentiation		
Low	10	24.4
Median	8	19.5
High	3	7.3
Unknown	20	48.8
TNM stage		
Ⅲb	3	7.3
Ⅳ	38	92.7
Prior treatment		
2	24	58.5
≥3	17	41.5
EGFR-TKI before		
Yes	24	58.5
No	17	41.5
ECOG: Eastern Cooperative Oncology Group; PS: performance status; EGFR: epidermal growth factor receptor; TKI: tyrosine kinase inhibitor.		

41例患者中NI方案主要作为三线、四线和四线以上治疗方案，分别为23例（56.1%）、12例（29.3%）和6例（14.7%）。一线治疗方案以铂类联合方案为主36例（87.8%），其中吉西他滨联合顺铂/卡铂方案21例，紫杉醇联合顺铂/卡铂方案9例，多西他赛联合顺铂/卡铂方案3例，培美曲塞联合顺铂/卡铂方案3例。其他患者因为EGFR基因突变阳性在一线治疗选择了EGFR-TKI治疗5例（12.2%）。二线治疗方案以单药多西紫杉醇为主，共22例（53.6%），其他包括单药培美曲塞8例（19.5%），多西他赛联合铂类5例（12.2%），EGFR-TKI 6例（14.6%）。三线治疗方案包括EGFR-TKI 9例，培美曲塞6例，多西他赛3例。四线治疗包括EGFR-TKI 6例。所有患者均未接受维持治疗。

### 2.2 化疗方案完成情况

41例患者共进行150周期化疗，其中23周期（15.3%）出现用药推迟或剂量调整。按计划每周期长春瑞滨给药2次，异环磷酰胺给药3次，长春瑞滨实际共用药286次，异环磷酰胺445次（表 2）。其中取消长春瑞滨第8天用药14例次，取消原因分别为血中性粒细胞下降8例次，纳差乏力2例次，感染4例次；取消第2或3天异环磷酰胺用药5例次，原因分别为中性粒细胞下降4例次，感染1例次。

**2:** NI方案完成及调整情况 Administation of NI chemotherapy

Cycle number	*n*	Treatments administrated			Treatments omited	
		NVB	IFO		NVB	IFO
1	41	77	121		5	2
2	33	65	99		1	0
3	27	51	78		3	3
4	20	37	60		3	0
5	15	30	45		0	0
6	14	26	42		2	0
Sum	150	286	445		14	5
NVB: Vinorelbine; IFO: Ifosfamide.						

### 2.3 疗效观察

41例患者中6例未进行疗效评价，包括1例患者在1周期化疗后死亡，5例患者1周期化疗后体能评分明显恶化，未评价疗效。其他35例患者进行了疗效评价，CR者0例，PR者3例（7.3%），SD者25例（61.0%），PD者7例（17.1%），不能评价疗效6例（14.6%）。ORR值为7.3%，DCR值为68.3%。3例部分缓解的患者均为腺癌，之前均接受过3个以上的方案治疗，包括含铂化疗，均完成6程NI方案化疗。

中位随访时间18个月（3.0-49.5），共28例患者死亡，39例出现疾病进展，中位无进展生存时间5.5个月（图 1）。单因素分析和多因素分析均显示PFS与各种临床因素均无统计学相关（表 3）。中位生存期为10.5个月，Kaplan-Meier生存曲线见图 2。单因素分析显示OS与病理类型和EGFR基因突变状态相关（表 4），但多因素分析显示OS与各种临床因素均无相关。

**1:**
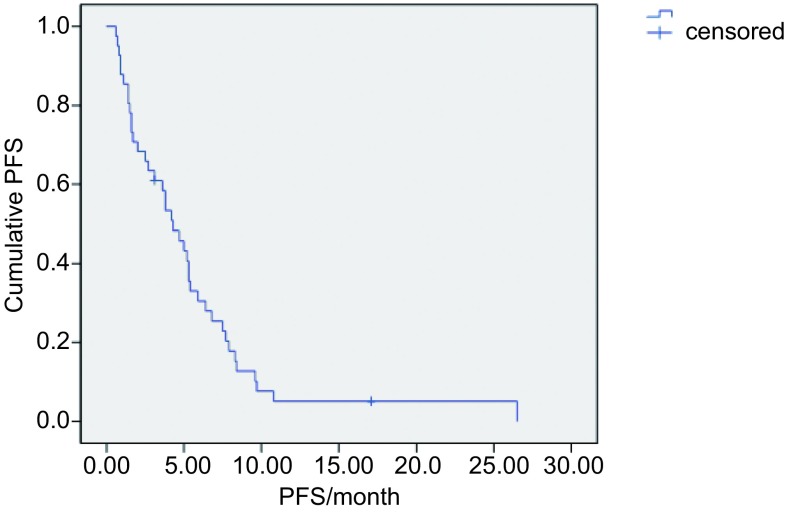
无疾病进展生存时间的*Kaplan-Meier*曲线 *Kaplan-Meier* curve of PFS

**3:** PFS时间的各种临床因素的单因素分析 Univariate analysis of PFS between clinical factors

Factor	*n*	PFS (mo)	Range	*χ*^2^	*P*
Gender				0.261	0.609
Male	27	5.2	3.4-7.0		
Famale	14	3.8	1.1-6.5		
Age (yr)				0.471	0.492
≥60	18	4.7	3.2-6.2		
< 60	23	3.8	1.5-6.1		
ECOG PS				0.558	0.455
0	29	3.8	0.6-7.0		
1-2	12	4.3	3.5-5.1		
Pathological type				3.417	0.065
Non-squamous	26	5.0	2.9-7.1		
Squamous	15	2.7	0-5.5		
Treatment line				0.409	0.522
3	24	3.8	2.1-5.5		
> 3	17	5.3	2.2-5.4		
EGFR-TKI before				0.115	0.735
Yes	24	5.0	3.7-6.3		
No	17	3.8	2.2-5.4		
*EGFR* gene mutation				4.065	0.131
Negative	11	7.9	3.6-12.2		
Positive	8	3.8	2.8-4.8		
Unknown	22	2.7	0-5.6		
PFS: progression free survival.					

**2:**
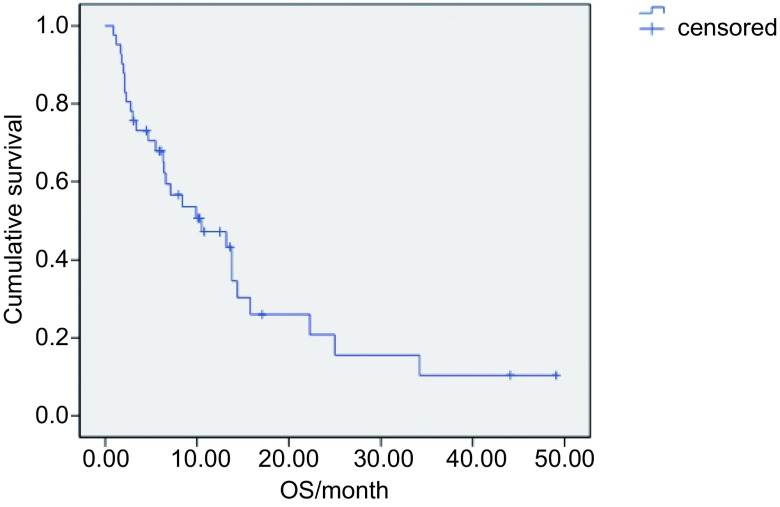
总生存期的*Kaplan-Meier*曲线 *Kaplan-Meier* curve of OS

**4:** OS时间的各种临床因素的单因素分析 Univariate analysis of OS between clinical factors

Factor	*n*	OS (mo)	Range	*χ*^2^	*P*
Gender				0.301	0.583
Male	27	8.4	3.2-13.6		
Famale	14	13.2	9.4-17.0		
Age (yr)				0.257	0.612
≥60	18	10.5	4.0-17.0		
< 60	23	9.9	3.4-16.4		
ECOG PS				0.018	0.892
0	29	13.8	6.6-21.0		
1-2	12	8.4	1.4-15.4		
Pathological type				4.084	0.043
Non-squamous	26	13.2	8.6-17.8		
Squamous	15	7.1	0.4-13.8		
Treatment line				0.026	0.872
3	24	8.4	0-17.8		
> 3	17	10.5	6.3-14.7		
EGFR-TKI before				1.350	0.245
Yes	24	13.2	10.1-16.3		
No	17	7.1	0.2-14.0		
*EGFR* gene mutation				8.182	0.017
Negative	11	13.8	0-30.5		
Positive	8	13.2	0.3-26.1		
Unknown	22	4.7	0-10.8		
OS: overall survival.					

### 2.4 不良事件及不良反应

记录150个用药周期发生的不良事件（表 5）。没有观察到药物不良反应导致死亡。血液系统毒性是常见的不良反应，包括血红蛋白下降、中性粒细胞下降、白细胞下降，但未观察到血小板下降。其他常见的非血液学不良反应包括恶心、呕吐、食欲减退、周围神经病、乏力，但3度/4度非血液学不良反应发生率很低。

**5:** 化疗相关不良事件 Adverse events of chemotherapy

Adverse event	All events			3/4 degree events	
	*n*	%		*n*	%
Decrease of hemoglobin	63	42.0		13	8.7
Neutropenia	40	26.7		16	10.7
Decrease of white blood cells	44	29.3		13	8.7
Anorexia	19	12.7		1	0.7
Nausea	24	16.0		2	1.3
Vomit	11	7.3		2	1.3
Peripheral neuropathy	16	10.7		0	0
Pain	24	16.0		1	0.7
Fatigue	13	8.7		1	0.7
Infection	9	6.0		7	4.7
Fever	8	5.3		0	0
Fever with neutropenia	4	2.7		4	2.7
Constipation	4	2.7		0	0
Diarrhea	1	0.7		0	0
Abnoral ALT	3	2.0		1	0.7
Pulmonary embolism	1	0.7		1	0.7
Ileus	1	0.7		1	0.7
ALT: alanine transaminase					

## 3 讨论

不能手术的进展或转移的晚期NSCLC中，目前推荐的一线治疗方案以含铂类双药联合治疗方案为主，二线治疗包括多西他赛和培美曲塞（在非鳞状NSCLC中）。EGFR-TKI适用于EGFR基因突变的患者和ALK抑制剂适用于ALK基因重排的患者，有相关基因改变的患者应尽早使用相应的靶向药物治疗^[1]^。患者在接受过2个化疗方案和靶向治疗后无标准可选择的治疗方案，但是多项临床试验中证实在这些患者中，特别是体能评分好的患者，进一步接受化疗可以延长患者的生存时间，包括患者之前未接受过的化疗药物或再次使用之前疗效好的药物^[2]^。而长春瑞滨在晚期NSCLC的一线和二线治疗中很少选用，但其副反应少，含长春瑞滨的治疗方案在NSCLC的三线和三线以后治疗中的作用值得探讨。

异环磷酰胺和长春瑞滨均为传统的化疗药物，在NSCLC中异环磷酰胺单药初治ORR达到20%-25%^[3]^，而长春瑞滨单药治疗ORR约30%。1995年，长春瑞滨联合异环磷酰胺方案作为不含铂类的双联化疗方案进入临床Ⅰ期试验^[4]^。多项国外临床试验和研究^[5-8]^显示长春瑞滨联合异环磷酰胺方案在初治晚期NSCLC患者中能获得较高缓解率，总缓解率ORR 30%-55%，中位OS 7.4个月-12.5个月。国内的研究^[9-11]^得到类似的结果，总缓解率40%-50%。然而NI方案在晚期NSCLC二线和二线以后治疗中的作用的研究少见。一项研究^[12]^比较不同剂量多西他赛与长春瑞滨或异环磷酰胺单药在铂类耐药的晚期NSCLC中的治疗，118例患者使用长春瑞滨或异环磷酰胺单药，PR 0.8%，SD 31.0%，中位PFS 7.9周，中位OS 5.6个月，这暗示长春瑞滨或异环磷酰胺单药治疗铂类耐药后NSCLC并未得到缓解，生存期亦没有得到改善。另一项研究^[13]^NI方案用于29例铂类治疗后的晚期NSCLC患者，PR仅1/29（3.4%），SD 9/29（31.0%），中位PFS 4.5个月，中位OS 10个月。这显示NI方案在铂类药物治疗后没有表现出明显的缓解率，但多数患者获得疾病稳定，并可能与较长的中位生存期相关。

本研究首次讨论NI方案晚期NSCLC三线和三线以后治疗中的疗效和安全性，其中38例患者曾经使用过铂类及多西他赛治疗。客观疗效评价显示ORR值为7.3%，DCR值为68.3%，与多西他赛或培美曲塞单药二线治疗NSCLC的疗效相似。特别是中位OS和中位PFS，与多西他赛或培美曲塞单药二线治疗NSCLC的疗效也相似。获得PR的3例患者均为腺癌，之前均接受过3个以上的方案治疗，包括含铂化疗，提示NI方案在铂类及多西他赛耐药的晚期NSCLC中的近期疗效可能与肿瘤病理分类相关，同时也提示有机会接受铂类和多西他赛治疗的患者，可能从三线长春瑞滨联合异环磷酰胺方案中受益。

本研究中NI方案化疗共进行150周期，其中15.3%出现中断、推迟或剂量调整，其主要原因为血中性粒细胞减少。血液系统毒性是主要不良反应，也是限制NI药物剂量的主要因素，这与既往研究^[5-11]^相符。3度/4度不良反应主要是中性粒细胞下降和粒细胞缺乏伴发热。其他3度/4度非血液学不良反应很少见，未观察到3度/4度周围神经病，没有观察到因药物不良反应导致的死亡。因此总体上患者耐受性好。

总之，长春瑞滨联合异环磷酰胺方案在晚期NSCLC三线及三线以上治疗中安全性较好，同时多数患者可获得一定疗效，但需要进一步大样本的临床试验来证实是否可以延长OS。
